# Treatment Outcomes and Factors Affecting Survival in Pediatric Acute Myeloid Leukemia

**DOI:** 10.1007/s00277-026-06906-4

**Published:** 2026-02-26

**Authors:** Yurday Öncül, Arzu Akyay, Bengü Macit, Ünsal Özgen

**Affiliations:** 1 Department of Pediatric Hematology and Oncology, Inonu University School of Medicine, Malatya, Türkiye; 2 Department of Pediatric Hematology and Oncology, Ondokuz Mayıs University School of Medicine, Samsun, Türkiye

**Keywords:** Acute myeloid leukemia, Pediatric, Prognosis, Risk stratification, Survival outcomes

## Abstract

This study examined the clinical, genetic, and treatment response characteristics of pediatric patients with acute myeloid leukemia (AML) treated according to the 2004 and 2012 AML-BFM protocols. This study aimed to determine survival outcomes and the factors affecting them, and to compare the results with international data. This study retrospectively evaluated pediatric AML patients aged < 18 years in eastern Turkey between January 2010 and December 2023. The AML-BFM 2004 and AML-BFM 2012 protocols were applied to the patients. The risk groups of the patients were determined based on their responses to chemotherapy and genetic results. Survival analyses were conducted using the Kaplan–Meier method. Univariate and multivariate Cox regression analyses were used. Forty-nine patients were included in this study. The median age at diagnosis was 12 years, and 51% of the patients were female. The most common morphological subtype was AML M3, accounting for 32.7% of cases. Complete remission was achieved in 81.6% of patients after induction therapy, whereas 12.2% died during this phase. Relapse occurred in 26.5% of patients. Overall, 38.8% of patients died. AML-M3 patients had a median overall survival (OS) of 113 months, which was better than that of non-AML-M3 patients. Five-year survival rates were 94% for AML-M3 patients and 43% for non-AML-M3 patients. Lower survival rates were observed in patients with leukocyte counts ≥ 50 × 10⁹/L, low hemoglobin levels, non-AML-M3, and poor induction response. Mortality was higher in patients younger than two years and > 14 years. There were no significant differences in mortality, survival, or relapse rates between the AML-BFM 2004 and AML-BFM 2012 protocols (*p* > 0.005). No significant association was observed between mortality and leukapheresis. Further research should focus on developing personalized treatments for high-risk patients and those without non-AML-M3, given their poorer survival rates than those of AML-M3 patients.

## Introduction

In recent years, survival rate of pediatric acute myeloid leukemia (AML) has increased to as high as 70–80% [1, 2]. Mortality rates have declined with the development of induction chemotherapy blocks and supportive care [3–7]. Improvements in conditioning regimens for hematopoietic stem cell transplantation and effective management of complications have enabled cure in the majority of patients [8, 9]. Despite the advent of novel agents and stem cell transplantation, the treatment of pediatric AML remains challenging.

Elucidation of the clinical, cytogenetic, and molecular heterogeneity of AML has led to the development of risk-adapted therapies for specific AML subgroups. In the AML-BFM 2004 protocol, patients are classified as SR and HR based on their treatment responses and genetic characteristics after the induction block. In the AML-BFM 2012 protocol, they are classified as SR, IR, and HR. The risk group classifications for the AML 2004 and AML 2012 protocols are provided in Table 1 [10]. In acute promyelocytic leukemia (AML M3), the use of chemotherapy-free regimens combining all-trans retinoic acid (ATRA) and arsenic trioxide (ATO) has enabled achievement of remission rates of approximately 90% [11–13].

**Table 1:** Risk group definitions

AML BFM 2004	SR	M1/M2 with Auer rods, AML with t(8;21), M4Eo with inv (16), M3 AML in Down’s syndrome
	HR	M0, M1/M2 without Auer rods M4, M5, M6, M7
AML BFM 2012	SR	t(8;21), inv (16), t (10;11), NPM1, CEBPαdm
	IR	Patients not qualified as SR group or HR group
	HR	t(9;22), t(4;11), t(5;11), t(10;11), t(6;9), t(7;12), der12p, isolated monosomy 7, FLT3-ITD-WT1mut, complex karyotype

There are studies on the role of minimal residual disease (MRD) (2). Identifying risk groups using MRD has made it possible to achieve higher survival rates [2].

The aim of this study was to retrospectively analyze the clinical and genetic characteristics of our patients diagnosed with AML, to evaluate treatment responses and survival outcomes under the AML-BFM 2004 and AML-BFM 2012 protocols, and to identify factors affecting survival by comparing our findings with international data.

## Materials & Methods

This study retrospectively reviewed 49 pediatric patients aged < 18 years who were diagnosed with AML at a tertiary university hospital in Turkey between January 2010 and December 2023. Demographic data, clinical findings at the initial diagnosis, use of leukapheresis, administration of cytoreductive therapy prior to induction, induction response, relapse status, outcomes of stem cell transplantation, and current status (alive/deceased) of these patients were evaluated. Age at diagnosis was stratified into three groups: <2, 2–13, and ≥ 14 years. Hemoglobin level (g/dL), leukocyte count (x10^9^/L), platelet count (x10^9^/L), and blast percentages in the bone marrow aspirate and peripheral smear (%) at diagnosis were recorded. Leukocyte counts were categorized as < 50 × 10^9^/L or ≥ 50 × 10^9^/L.

The diagnosis of AML was established by morphological classification of the bone marrow aspirate and immunophenotyping using flow cytometry. Cytogenetic and molecular genetic analyses were performed using fluorescence in situ hybridization (FISH) and quantitative real-time PCR. Cerebrospinal fluid was obtained due to central nervous system involvement.

**Fig. 1:**
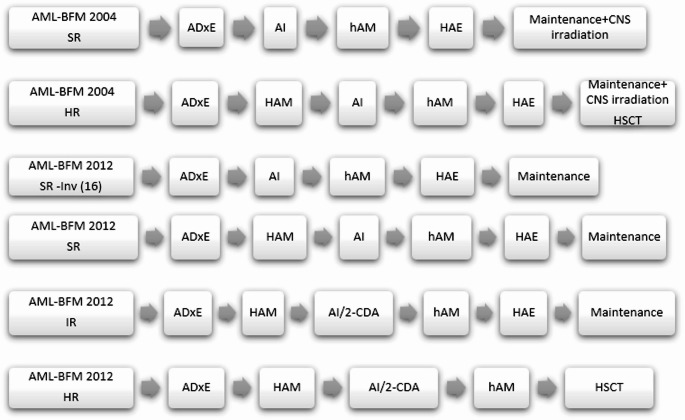
Treatment protocols. SR: standard risk; HR: High risk; IR: İntermediated risk; AI: Cytarabine/ Idarubicin; haM: Cytarabine/Mitoxantrone, HAE: high dose cytarabine/Etoposide. 2-CDA: 2-chloro-2-deoxyadenosine. CNS: Central nervous system, HSCT: Hematopoetic stem cell transplantation

The patients were treated according to the AML-Berlin–Frankfurt–Munster 2004 and AML-BFM 2012 protocols. In the AML-BFM 2004 protocol, patients are stratified into standard-risk (SR) and high-risk (HR) groups based on their treatment response after induction and genetic features at the time of initial diagnosis. The AML-BFM 2012 protocol also stratifies risk as SR, intermediate-risk (IR), and HR. In the AML-BFM protocols, after the administration of ADxE (cytarabine/liposomal daunorubicin/etoposide) or AIE (cytarabine/idarubicin/etoposide) during the first induction phase, randomization was performed. Chemotherapy regimens were administered every 28 days to the SR, IR, and HR groups (Fig. 1) [3]. For children under 12 months old and weighing less than 10 kg, dose adjustments were made per kilogram. Hematopoietic stem cell transplantation (HSCT) indication was determined for high-risk patients.

In addition, patients were divided into two groups: AML M3 (acute promyelocytic leukemia) and non-AML M3 (AML M1, M2, M4-7, biphenotypic, undifferentiated type. Mortality rates and survival rates were examined separately in three groups: AML M3, non-AML M3, and Down syndrome.

The AML-BFM 2004 protocol was used between 2010 and 2015 and the AML-BFM 2012 protocol was used between 2016 and 2023. Treatment efficacy was assessed at the morphological level by bone marrow aspiration at the end of the chemotherapy blocks. Complete remission was defined as normocellular marrow with ≤ 5% blasts, peripheral neutrophil count ≥ 1 × 10^9^/L, platelet count ≥ 80 × 10^9^/L, absence of extramedullary disease, and no blasts in the central nervous system.

The ATRA and chemotherapy protocol was administered to AML-M3 patients. For two patients, only ATRA + CT and ATO were given during the induction phase. During induction therapy, ATRA was administered for 14 days, followed by a 7-day break, and then administered again for 14 days, making a total of 4 cycles. In maintenance therapy, a total of 3 cycles of ATRA were given for 14 days every 3 months. Arsenic trioxide (ATO) therapy is used alternately with ATRA in relapsed AML M3 patients. In our study, ATO and ATRA treatment were administered together to two of our patients with AML-M3. The patients who received ATO were not in relapse. The other AML M3 patients received classical chemotherapy and ATRA.

### Statistical Analysis

Data are expressed as mean ± SD or median (IQR) based on normality. Intergroup comparisons were performed using the chi-squared test, Fisher’s exact test, and t-tests. Non-normally distributed data were analyzed using the Mann–Whitney U test. Survival rates were calculated using the Kaplan–Meier method. Survival analyses were conducted using univariate analysis. Cox regression analysis was performed as both univariate and multivariate analyses. Receiver operating characteristic (ROC) curve analysis was used to evaluate prognostic prediction performance. Statistical analyses were conducted using the IBM SPSS Statistics Data Editor. Statistical significance was set at *P* < 0.05.

## Results

A total of 49 patients met the inclusion criteria.

### Demographic and Clinical Characteristics

The median age at diagnosis was 12 years (IQR;6–14), with the most frequent age group being 2–13 years. Twenty-five patients (51%) were female. The median duration of pre-diagnosis symptoms was 10 days (IQR: 5–17) days. The most common presenting complaints were fever and bleeding (54% and 42%, respectively). The most frequent physical examination findings were petechiae/ecchymosis and hepatosplenomegaly (44%) and 34%, respectively). Down syndrome was the most common comorbidity, present in 5 patients (10.2%). Central nervous system (CNS) involvement was observed in 5 patients (10.2%). The demographic characteristics and clinical findings are summarized in Table 2.

**Table 2:** Demographics and general clinical data of patients

Variable		No.of patients (%) ( *n* = 49)	
Age (years)*		12 years (6–14)	
< 2		5 (10.2)	
2–13		31 (63.3)	
≥ 14		13 (25.5)	
**Sex (female/male)**		25 (51)/24 (49)	
**Duration of complaints before diagnosis in days (median**,** IQR)**		10 days, (5–17)	
**Presenting symptoms**		**Physical examination findings**	
Fever	27 (54)	Petechiae ecchymosis	22 (44)
Bleeding	21 (42)	Hepatosplenomegaly	17 (34)
Weakness, fatigue	13 (26)	Gingival enlargement	1
Abdominal pain	8 (16)	Lymphadenopathy	10
Extremity and joint pain	6 (12)	Hepatomegaly	5
Respiratory tract complaints	6 (12)	Splenomegaly	3
Visual impairment	1 (2)	Neurological findings	1
Without complaints	1 (2)	FM normal	3
**Type of comorbidity** (*n* = 11)		11 (22.4)	
Down syndrome		5 (10.2)	
MDS		2 (4.1)	
Fanconi AA		1 (2)	
Epilepsy		1 (2)	
JMML		2 (4.1)	
Type I DM		1 (2)	
**Leukocyte count (x10** ^**9**^ /L)*		27.9 (4.2–91)	
< 50 × 10^9^/L		29 (59.2)	
≥ 50 × 10^9^/L		20 (40.8)	
Hemoglobin (g/dL)**		8.1 ± 2.1	
Platelet count (x10^9^/L)*		35 (19.5–50.5)	
**CNS involvement**		5 (10.2)	
**Extramedullary infiltration**		7 (14.2)	

*median (IQR), **mean ± SD

### Biochemical Characteristics

According to the French-American-British (FAB) classification, the most frequent morphological subtype was AML M3 (APL) in 16 cases (32.7%) (Table 3). The median leukocyte count was 27.9 × 10^9^ /L (IQR, 4.2–91), and 20 patients (40.8%) had leukocyte counts ≥ 50 × 10^9^/L. The mean hemoglobin level was 8.1 ± 2.2 g/dL, and the median platelet count was 35 × 10^9^ /L (IQR, 19.8–50.2) (Table 2). The mean blast percentage in peripheral smears at diagnosis was 50 ± 36.5%, while bone marrow aspiration revealed a mean blast percentage of 76.3 ± 21.4%.

**Table 3:** Clinical characteristics of pediatric AML patients

Variable	No.of patients (%) (*n* = 49)
FAB classification	
M0	1 (2)
M1	2 (4.1)
M2	7 (14.3)
M3	16 (32.7)
M4	8 (16.3)
M5	8 (16.3)
M6	1 (2)
M7	2 (4.1)
Undifferentiated Type	3 (6.1)
Biphenotypic	1 (2)
**Risk degree**	
Standart-risk	28 (57.2)
Intermediate-risk	6 (12.2)
High-risk	15 (30.6)
**Leukapheresis**	10 (20.4)
**Cytoreductive treatment**	22 (44.9)
**AML-BFM 2004** **(2010–2015)**	17 (34.7)
**AML-BFM 2012** **(2016–2023)**	32 (65.3)
**Relapse**	13 (26.5)
AML M1,2,4,5,6,7	12 (24.5)
AML M3	1 (2)
**Death**	19 (38)
AML M1,2,4,5,6,7	18 (36.7)
AML M3	1 (2)

^a^Some patients have multiple complaints

^b^Cough, shortness of breath, chest pain

Risk stratification classified 28 patients (57.2%) as SR, six (12.2%) as IR, and 15 (30.6%) as HR.

The most frequent genetic mutation observed was t(15;17) in 16 patients (32.6%), followed by t(8;21) in 9 patients (18.3%). Other genetic mutations are listed in Table 4.

**Table 4:** Genetic mutations in pediatric AML patients

Genes*	Total number of patients *N* (%)
t(15;17) PML/RARA	16 (32.6)
t(8;21) AML 1/ETO	9 (18.3)
11q23 (MLL)	3 (6.1)
İnv (16)	3 (6.1)
Monosomy 5, monosomy 7 Trisomy 7	2 (4)
t(9;22) (q34;q11.2)	3 (6.1)
t (4;11)	2 (4)
NRAS	2 (4)
17p 13 deletion	1 (2)
FLT3	3 (6.1)
WT1	2 (4)
not performed	2 (4)
Normal	10 (20.4)

*Some patients have multiple gene mutations

### Treatment Outcomes

Seventeen patients (34.7%) were treated using the AML-BFM 2004 protocol, and 32 under the AML-BFM 2012 protocol. Cytoreductive therapy was administered to 22 patients (44.9%), and leukapheresis was performed in 8 (16.3%), with all leukapheresis patients achieving a response. All patients received the ADE (cytarabine, daunorubicin, etoposide) protocol as initial therapy. Complete remission (bone marrow blasts < 5%) after induction was achieved in 40 patients (81.6%). Six patients (12.2%) died during induction. Six patients underwent bone marrow transplantation (BMT), of whom three (50%) died due to post-transplant relapse (Table 3).

### Survival Outcomes

Thirteen patients (26.5%) experienced relapse: One in the AML M3 group and 12 (24.5%) in the non-AML M3 group. The mean time-to-relapse from initial diagnosis was 10.4 ± 5.2 months. All patients with relapse received IDA-FLAG therapy (idarubicin, fludarabine, and cytarabine). Twelve relapsed patients (92.3%) died, with a mean time to death after relapse of 1.5 ± 1.3 months. Among the BMT recipients, one patient underwent transplantation for HR disease without prior relapse, but later died due to post-transplant relapse. All relapsed patients who did not undergo BMT (*n* = 9) died (100%), whereas 3 of 4 BMT recipients (75%) died.

There was a patient with non-relapse mortality. In the AML M3 group, one patient with Down syndrome relapsed and succumbed to a pulmonary infection.

Overall, 19 patients (38.8%) died.

**Fig. 2:**
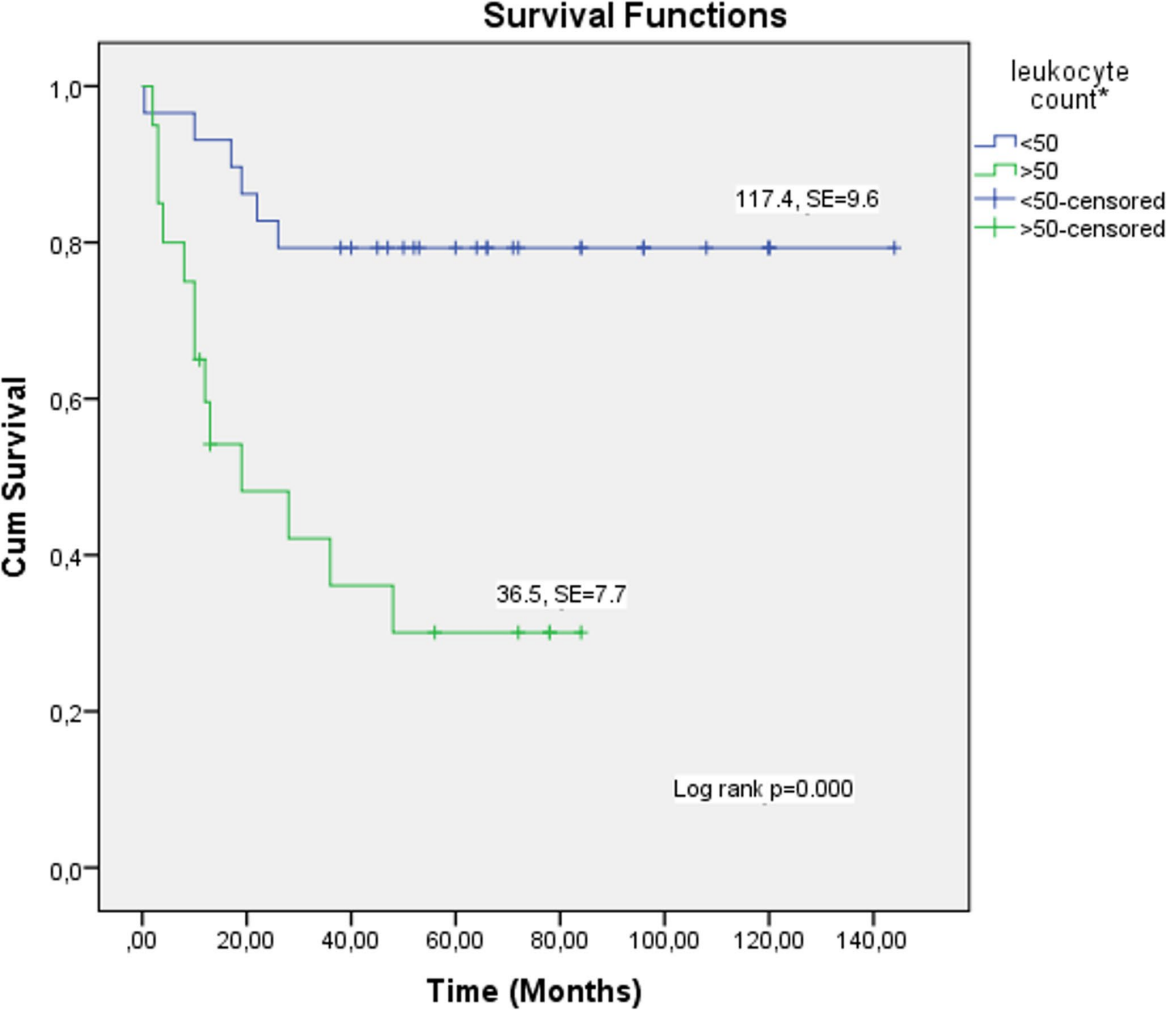
Kaplan–Meier analyses for OS rates of hyperleukocytosis patients

**Fig. 3:**
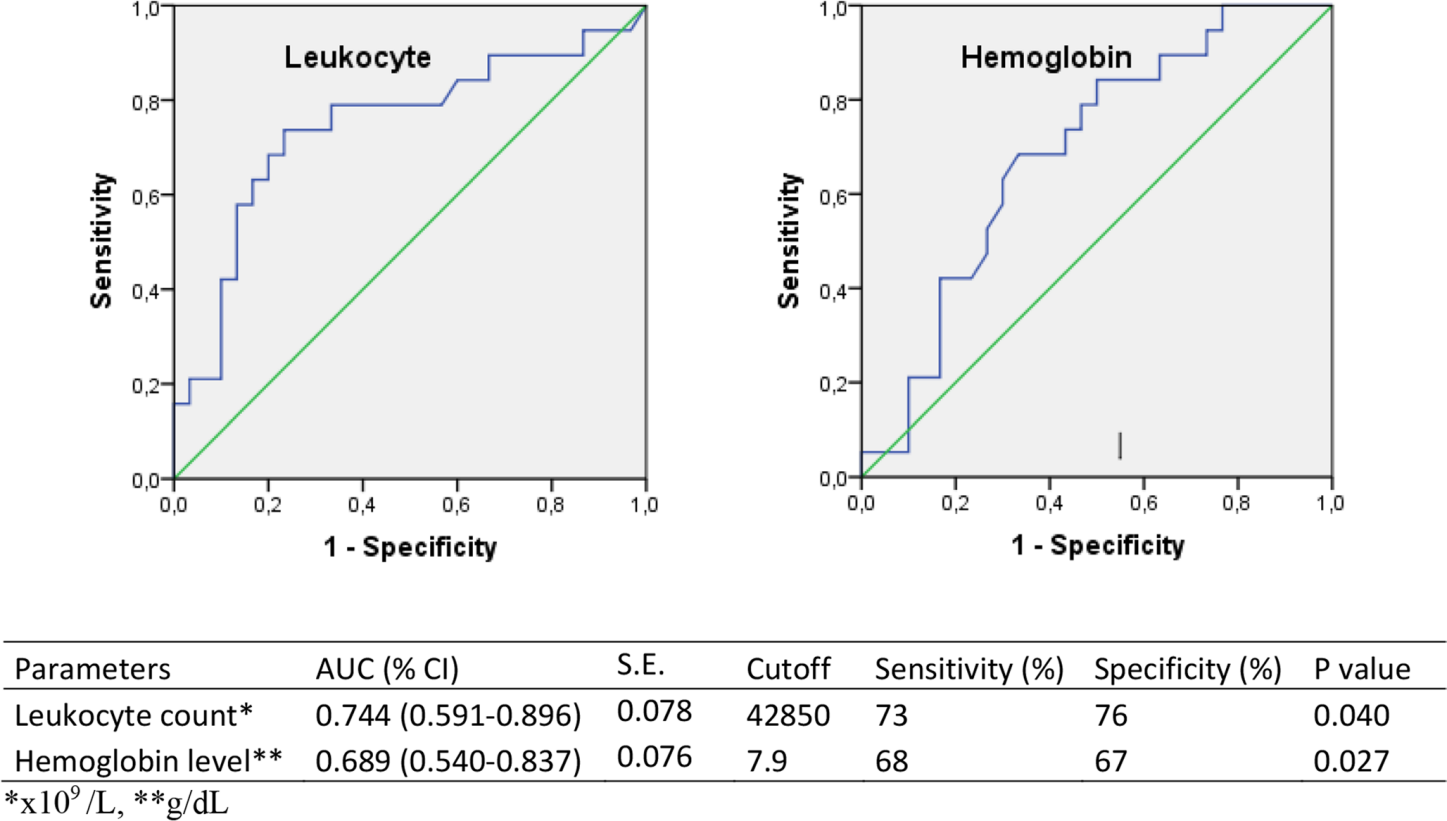
Receiver operating characteristic curves of the leukocyte count and hemoglobin level

No cases of hyperleukocytosis-related tumor lysis syndrome (TLS) were observed. Survival rates were significantly lower in patients with leukocyte counts ≥ 50 × 10^9^/L than in those with leukocyte counts < 50 × 10^9^/L (*P* = 0.002) (Fig. 2). ROC curve analysis revealed an area under the curve (AUC) of 0.744 for leukocyte count (*p* < 0.005), with a cut-off value > 42,850 × 10^9^ /L (Fig. 3). Lower hemoglobin levels at diagnosis were associated with reduced survival (*P* = 0.030) (Fig. 2). Low hemoglobin level at the time of diagnosis was identified as a negative prognostic factor for survival.

ROC curve analysis revealed an AUC value of 0.689 for the hemoglobin level, with a cut-off value of 7.9 g/dL (Fig. 3). Mortality was higher in patients receiving cytoreductive therapy, HR patients, non-AML M3 patients, and those with a poor induction response (*p* < 0.005) (Table 5).

**Table 5:** Relationship between clinical characteristics and mortality in patients

Variable	Alive *n* (%)	Death *n* (%)	*P* value
Age at diagnosis *			
< 2 years	2 (4.1)	3 (6.1)	0.05
2–13 years	23 (46.9)	8 (16.3)	
> 13 years	5 (10.2)	8 (16.3)	
Sex (female/male)	16 (32.7) /14 (28.6)	9 (18.4) /10 (20.4)	0.684***
Comorbidity	4 (8.2)	7 (14.3)	0.081**
CNS involvement	4 (8.2)	1 (2)	0.636**
Leucocyte count			
< 100 × 10^9^ /L	27 (55.1)	11 (22.4)	0.014**
> 100 × 10^9^ /L	3 (6.1)	8 (16.3)	
Hemoglobin g/dL (mean ± SD)	8.6 ± 2.2	7.3 ± 1.7	0.030*
**Cytoreductive treatment**	8 (16.3)	14 (28.6)	0.001***
**Leukapheresis**	3 (6.1)	5 (10.2	0.233**
Disease stage			
SR	23 (46.9)	5 (10.2)	0.002**
IR	2 (4.1)	4 (8.2)	
HR	5 (10.2)	10 (20.4)	
FAB diagnosis			
AML M3	15 (30.6)	1 (2)	0.002**
Non M3	12 (24.5)	16 (32.7)	
Down syndrome	3 (6.1)	2 (4.1)	
Auer rod	5 (10.2)	4 (8.2)	0.720**
Induction response			
Good	28 (57.1)	12 (24.5)	0.019**
Poor	2 (4.1)	7 (14.3)	
BMT	3 (6.1)	3 (6.1)	0.665**
Duration of symptoms	14.3 ± 12.4	10.7 ± 8.9	0.284*
Relapse	1 (2)	12 (25)	0.000**
Blast percentages in peripheral smear	44.5 ± 38.2	58.9 ± 32.7	0.180*
Blast percentages in bone marrow	78.2 ± 22.4	58.9 ± 32.7	0.444*

*T-test

**Fisher’s Exact test

***Chi kare

**Fig. 4:**
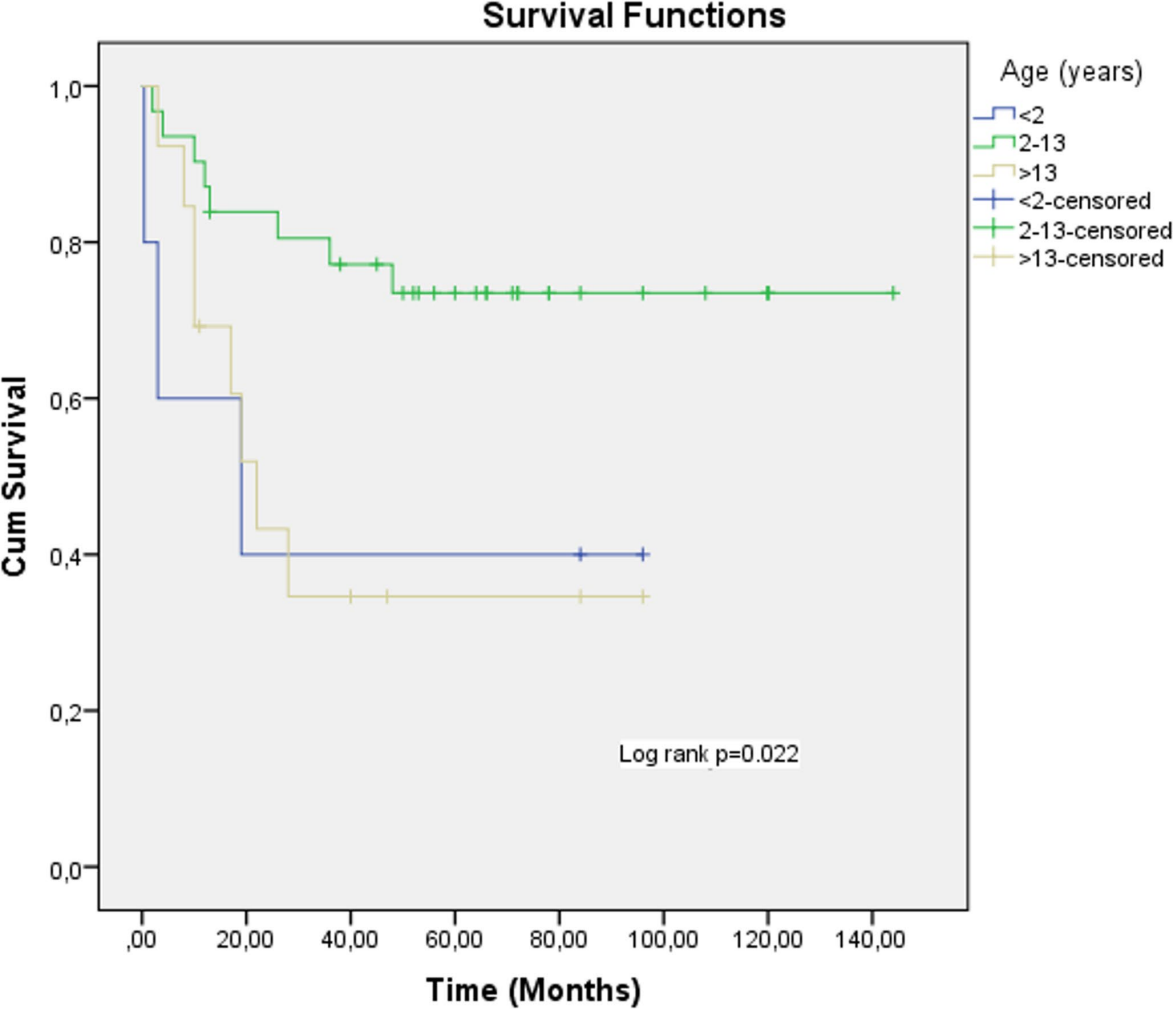
Kaplan-Meier analyses for OS rates of age

Mortality rates were elevated in children aged < 2 or ≥ 14 years at diagnosis (*p* = 0.05), whereas improved survival was observed in the 2–13-year age group (*p* = 0.022) (Fig. 4; Table 6).

**Table 6:** Factors affecting overall survival in pediatric AML patients

Variables	1-year cumulative proportion surviving at the time (%)	5-year cumulative proportion surviving at the time (%)	Overall OS (ay)	Log rank *P* value
**Age**				0.022
** < 2**	40 ± 22	40 ± 22	19 ± 17.5 (95% CI: 0.00-53.3)***	
** 2–13**	87 ± 06	73 ± 0.8	110 ± 10.1 (95% CI: 91.2-130.7)**	
** > 13**	60 ± 13	34 ± 14	22 ± 4.1 (95% CI: 13.9–30.1)***	
**AML M3**	94 ± 6	94 ± 6	113 ± 6.8 (95% CI: 99.3-126.3)**	0.009
**Non AML M3**	54 ± 9	42 ± 9	28 ± 15.2 (95% CI: 0.00-57.9)***	
**Down syndrome**	80 ± 18	53 ± 3	61.2 ± 18.6 (95% CI: 24.7–97.6)**	
**Relapse time**	%7±%7	0	3 ± 89 (95% CI: 1.2–4.7)***	0.000
**< 6 months**	0	0	1 ± 0	
**> 12 months**	%25±%21	0	2 ± 4.5 (95% CI: 0.0-10.8)***	
**Induction response**				0.000
** M1**	87 ± 5	69 ± 7	105.5 ± 9.2 (95% CI: 87.3-123.7)**	
** M3**	33 ± 16	16 ± 14	19.3 ± 7.3 (95% CI: 5.0-33.6)**	
**Disease stage**				
**AML-BFM 2004**				
**SR**	91 ± 8	73 ± 13	109.8 ± 16.9 (95% CI:76.6–143.0)**	
**HR**	60 ± 22	20 ± 18	28 ± 9.8 (95% CI:8.6–47.3)***	
**AML-BFM 2012**				0.000
** SR**	100 ± 0	87 ± 9	87.8 ± 5.3 (95% CI: 77.3–98.4)**	
**IR**	33 ± 19	33 ± 19	10 ± 4.0 (95% CI: 3.3–22.6)***	
**HR**	60 ± 15	38 ± 16	13 ± 4.9 (95% CI: 2.0-17.9)***	
**Leukocyte count**				0.000
**< 50 × 10** ^**9**^ **/L**	83 ± 7	79 ± 8	117.4 ± 9.6 (95% CI: 98.5-136.4)**	
**> 50 × 10** ^**9**^ **/L**	47 ± 12	30 ± 11	19 ± 10.5 (95% CI: 0-39.7)***	
**CNS involvement**				0.486
**Evet**	75 ± 22	75 ± 22	62.7 ± 13.2 (95% CI: 36.8–88.6)**	
**Hayır**	77 ± 6	58 ± 7	90.5 ± 9.7 (95% CI: 71.5-109.5)**	
**Protocol**				
**BFM-AML-2004**	81.9 ± 9	56 ± 12	90.7 ± 15.3 (95% CI: 60.6-120.7)**	0.924
**BFM-AML-2012**	75 ± 7	62 ± 8	64.2 ± 7.2 (95% CI: 49.9–78.5)**	

**mean, ***median

No significant association was found between mortality and sex, presence of comorbidities, CNS involvement, leukapheresis application, Auer rod presence, duration of pre-diagnosis symptoms, BMT, or blast percentages in the bone marrow and peripheral smears (*p* > 0.005, Table 5).

**Fig. 5:**
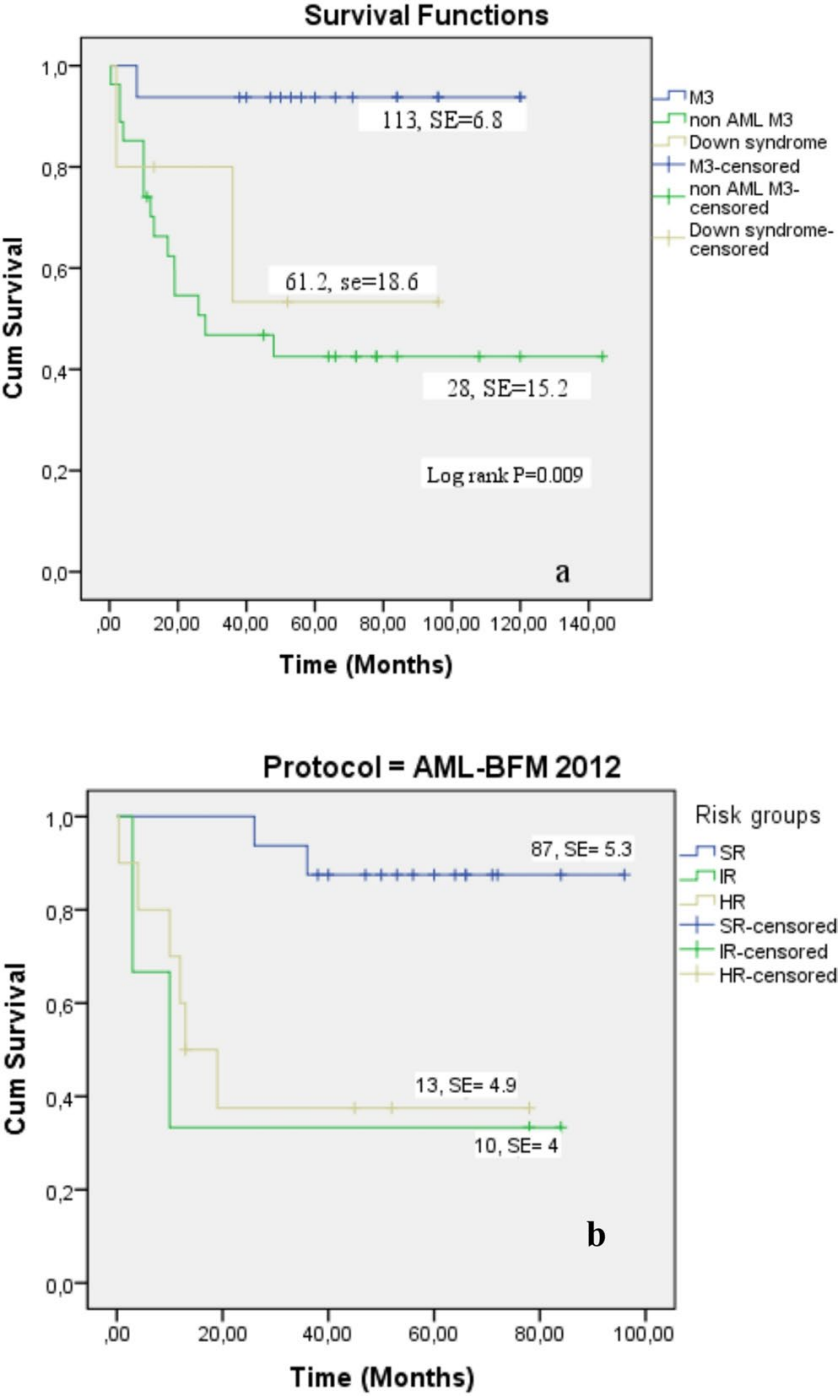

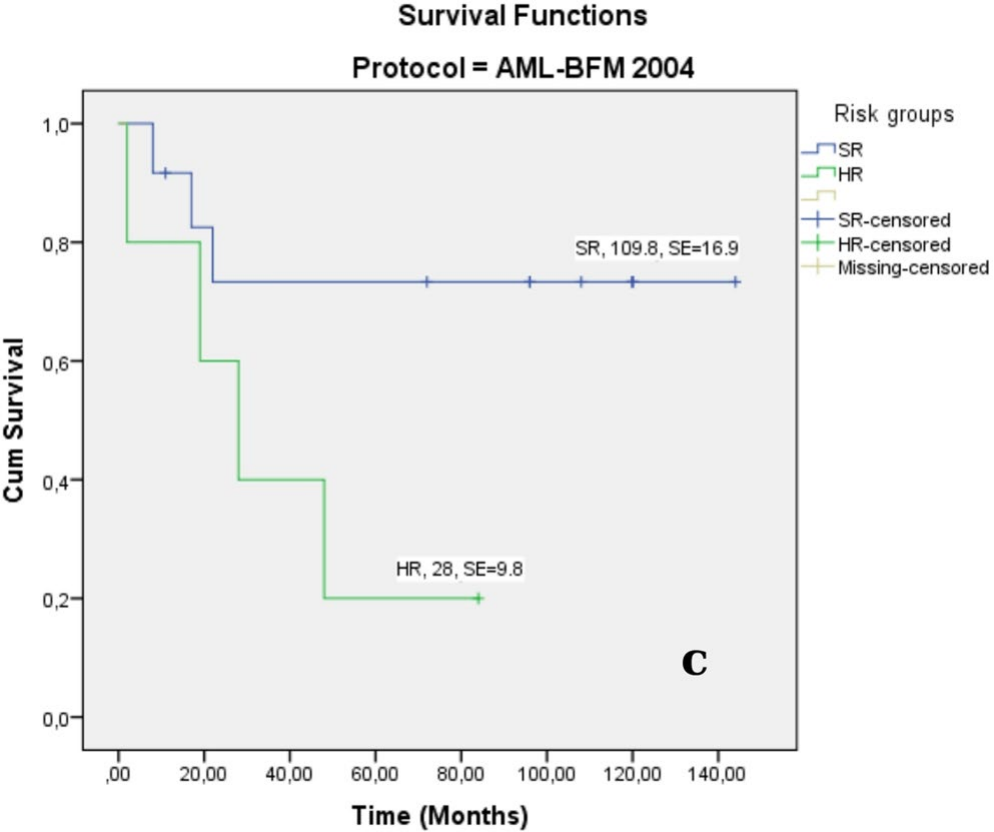
Kaplan–Meier analyses for OS rates of AML. OS rate of AML subtypes (**a**) and OS rate of AML risk groups for AML-BFM 2012 (**b**), and AML-BFM 2004 protocols (**c**)

The mean overall survival (OS) for AML-M3 patients was 113 months ±%6.8 months (95% CI: 99.3-126.3) (Fig. 5A), whereas the median OS for non-AML M3 patients was 28±%17.2 months (95% CI: 0.00-61.8) (*p* = 0.009, Table 6; Fig. 5A) months. The survival duration was shorter in the IR and HR groups than in the SR group (Fig. 5b, c).

The cumulative survival rates at 1 and 5 years were significantly longer in AML-M3 patients than in non-AML M3 patients. For AML-M3, cumulative survival was 94%±%6 at both time points. In non-AML M3 patients, 1-year and 5-year cumulative survival rates were 73% and 43%, respectively (*p* = 0.002, Table 6).

In relapsed patients, median OS after relapse was 3 ± 0.8 months (95% CI:1.23–4.76) (*p* = 0.000, Table 6).

**Fig. 6:**
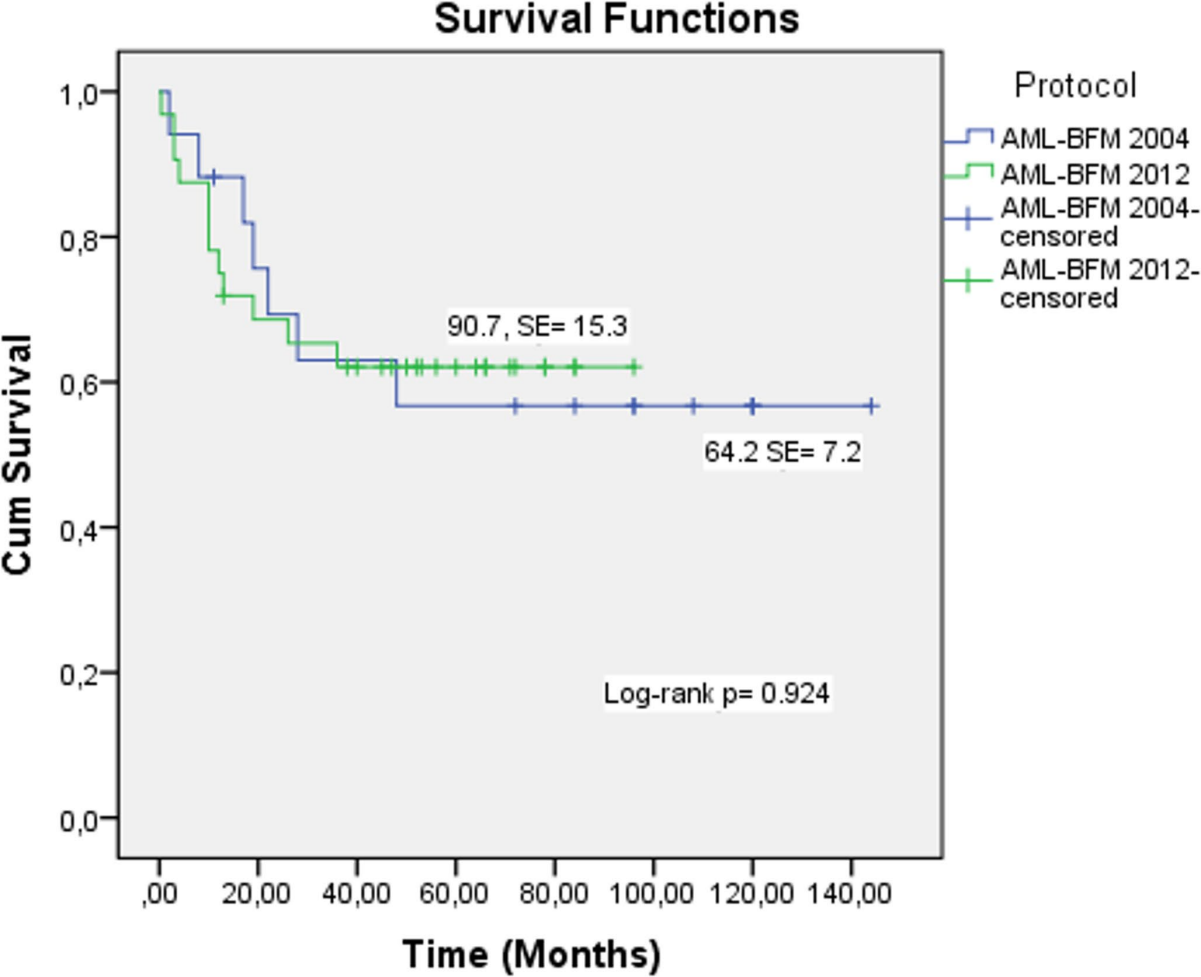
Kaplan–Meier analyses for OS rates of AML-BFM 2004 and AML-BFM 2012 protocols

No significant difference in survival rate was observed between the AML-BFM 2004 and AML-BFM 2012 protocols. The response rates were similar between the two protocols (Fig. 6).

### Impact of Disease Stage on Relapse

Relapsed patients with a lower disease burden at initial diagnosis had higher survival rates. Relapse occurred in 5 SR (10.2%), 2 IR (4.1%), and 6 h (12.2%) patients. Among patients with relapse, OS was lower in the IR and HR groups than in the SR group (*p* = 0.001). The median OS after relapse was 1 month for those who relapsed within the first 6 months and 2 ± 4.5 months for those who relapsed after 12 months (*p* = 0.000, Table 6).

Among the five patients (10.2%) with Down syndrome in our cohort, two died due to infection and chemotherapy toxicity, both of whom carried the t(8;21) mutation. The remaining three patients remained disease-free.

### Comparison of AML-BFM 2004 and AML-BFM 2012 Protocols

No statistically significant differences in mortality rates were observed between protocols (*p* = 0.801). Five-year OS was 56 for AML-BFM 2004 and 62 for AML-BFM 2012, with overall survival rates remaining comparable (*p* = 0.924) (Fig. 6) (Table 6). Relapse occurred in six patients (12.2%) treated under AML-BFM 2004 and seven patients (14.3%) treated under AML-BFM 2012. There was no significant difference between the protocols in terms of relapse rate (*p* = 0.331).

In univariate Cox regression analysis, it was found that patients who were < 2 years or > 13 years old, those who were non-AML M3, those with early relapse, those with an induction response of M3, IR and HR patients, and those with a leukocyte count of 50 × 10^9/L played a significant role in mortality. However, in multivariate Cox regression analysis, only patients with a short relapse time played a significant role in mortality (Table 7).

**Table 7:** Predictors of overall survival by Cox regression, among diagnosed AML patients

	Univariate analysis			Multivariate analysis		
	*P* value	Exp (B)	95% CI for Exp(B)	*P* value	Exp (B)	95%CI for Exp(B)
Age						
< 2 years	**0.035**			0.204		
2–13 years	0.933	1.059	0.28–4.01	0.149	0.27	0.05–1.60
> 13 years	**0.018**	0.30	0.11–0.81	0.994	1.01	0.20–4.98
**AML M3**	0.052			0.177		
**Non AML M3**	**0.016**	12.18	1.60-92.49	0.891	0.84	0.70-10.05
**Down syndrome**	0.079	8.60	0.78-95.00	0.220	6.48	0.33-128.06
**Relapse time**	**0.000**	0.92	0.88–0.95	**0.004**	0.89	0.82–0.96
**Induction response** M1 M3	**0.000**	5.81	2.25–15.06	0.720	0.735	0.14–3.96
**Disease stage**						
SR	**0.003**			0.296		
IR	**0.005**	6.80	1.81–25.65	0.320	2.71	0.38–19.26
HR	**0.002**	5.67	1.92–16.72	0.763	0.78	0.16–3.93
**Leukocyte count**						
**<** 50 × 10^9^/L	**0.002**	4.85	1.83–12.88	0.534	1.56	0.39–6.29
> 50 × 10^9^/L						

CI: Confidence interval

## Discussion

This study presents the clinical findings and treatment outcomes of pediatric patients with AML who were followed up at a tertiary hospital serving a large region in eastern Turkey. AML-M3 (APL) patients demonstrated excellent outcomes, particularly with the use of all-trans retinoic acid (ATRA) and arsenic trioxide (ATO). However, high mortality rates persisted in non-AML-M3 patients, especially in those with relapsed disease. No differences in mortality rate, OS, or relapse were observed between the AML-BFM 2004 and AML-BFM 2012 protocols. Factors influencing OS in our cohort included age, risk group, induction response at the initial diagnosis, and time-to-relapse after diagnosis.

There were 49 patients in a 14-year period. After 2020, there was a decline in the total number of patients at our clinic. The reason for this was that, due to technical inadequacies, our patients were referred to other centers for a certain period. Additionally, because of the severe earthquake that occurred in our region in February 2023, there was a significant decrease in our patient numbers until December 2023 (for about one year). Before 2020, there had not been any changes in the number of patients we followed up at our clinic.

The median age of our patients was notably higher than that reported in the literature; our cohort had a median age of 12 years, whereas a published series reported median ages between 5 and 10.5 years [14–16].

CNS involvement in pediatric AML varies between 6% and 16.4% [14, 17]. Previous studies have reported no significant impact of CNS involvement on survival [14, 17, 18], which is consistent with our findings.

Down syndrome is associated with an elevated risk of leukemia [19]. Although most Down syndrome-associated AML cases are classified as standard risk, mortality often results from treatment toxicity or infection. Relapsed/refractory AML in Down syndrome patients has a poor prognosis, with 3-year OS rates as low as 17%–26% [20, 21]. Recent advances have improved OS by approximately 93% in this population [22]. In our study, two Down syndrome died due to drug toxicity and infection, whereas none experienced relapse. The overall OS for Down syndrome patients in our cohort was 53%, lower than the rates reported in the literature.

The most common AML subtypes in pediatric patients are AML-M2 and AML M3, with AML M3 incidence ranging from 13% to 28.9% [14]. The incidence of AML M3 varies according to geographic regions. In most case series, it is observed at a rate of 2–10% [23]. It has been detected at the highest levels in Central and South America and in Italy [24]. In the present study, AML M3 was the predominant subtype.

The introduction of ATRA and ATO has significantly improved survival in AML M3, with 3-year survival rates reaching 91% [25–27]. However, survival rates remain suboptimal in developing countries [28]. In recent years, the overall survival rate of AML M3 patients treated with ATRA and ATO has increased to 99% [29, 30]. Historically, early mortality in AML M3 has been driven by coagulopathies occurring during induction or prior to treatment initiation [31]. The integration of ATRA into therapy has reduced hemorrhage-related deaths in AML M3 patients, which previously accounted for 10%–30% of fatalities [32]. None of the AML-M3 patients died of coagulopathy. Early diagnosis and prompt initiation of treatment, with appropriate management of coagulopathy, may reduce early mortality. The OS rate of our patients with AML M3 was consistent with that reported in the literature.

Non-AML M3 exhibited lower survival rates than those with AML M3. Survival rates in pediatric AML vary by sex (worse in males), age at diagnosis (lower in infants and adolescents), cytogenetic subtypes, and geographic disparities in treatment access and supportive care [33]. In India, median OS and event-free survival (EFS) for pediatric AML were reported as 12.6% and 14.6%, respectively. another study, AML M3 OS and EFS rates were 69.2% and 67.7%, than 45.3% and 36.7% in non-AML M3 cases [13, 28, 34, 35]. A 2008 Indian study reported a 5-year OS of 30% [36]. Data from a Brazilian study showed a 5-year OS of 49.7 ± 5.2% overall and 36.1 ± 11.2% in cytogenetically undefined AML [37]. In Europe, five-year survival rates for pediatric AML range from 67% to 73%, and five-year OS for AML M3 has been reported as 95% [38, 39].

Age at diagnosis is a critical risk factor for survival in pediatric patients with AML. Studies have indicated poorer survival outcomes in infants and adolescents [39]. A Dutch study reported an OS of 84% in children aged 1–9 years versus 66% in older children and adolescents (10–17 years) [40]. In our cohort, 5-year median OS was 94 for AML M3 and 42 for non-AML M3, and 53 for Down syndrome patients with an overall OS of 57 across all cases.

Consistent with previous reports, survival was lower in patients aged < 2 or ≥ 14 years at diagnosis. The median age of our patients was higher than those reported in other studies. Survival rates for our AML M3 patients were comparable to those from European reports; however, the OS in non-AML M3 cases was low. This may be attributed to the higher median age of the patient population.

Although treatment responses in the SR group of pediatric AML improved over time, the mortality rates in the intermediate and HR groups remained high. In our study, the median survival time in the HR group was lower than that in the SR group. Notably, among the patients treated according to the AML-BFM 2012 protocol, survival in the IR group was as poor as that in the HR group. Therefore, we emphasize that patients with IR warrant the same level of careful evaluation as those in the HR category.

In pediatric AML, hyperleukocytosis–associated leukostasis and bleeding increases the risk of early mortality and morbidity. It is well established that a high leukocyte count at diagnosis adversely affects survival [41]. In the present study, patients with elevated leukocyte counts had shorter survival times. TLS due to hyperleukocytosis is less common in AML [42]; in fact, none of our patients developed TLS related to hyperleukocytosis. In our study, higher mortality was detected in patients receiving cytoreductive therapy. Since the risk of developing tumor lysis syndrome in AML patients is low, starting treatment with full-dose chemotherapy instead of cytoreductive therapy may reduce the mortality rate. However, further research is needed on this subject.

Leukapheresis is used to rapidly reduce leukocyte burden and mitigate TLS risk [43]. The 2016 American Society for Apheresis guidelines recommend prophylactic or therapeutic leukapheresis for patients with WBC counts > 100,000/µL [43]. However, recent studies on pediatric AML suggest that early initiation of induction therapy in hyperleukocytosis may be preferable to leukapheresis [44]. In our cohort, leukapheresis had no effect on survival. Furthermore, leukapheresis is not universally available and referral to other centers for the procedure can delay treatment initiation.

Relapse rates in pediatric AML have been reported between 26% and 48% [36]. The initial risk classification has been linked to survival after relapse [21]. For example, in the Children’s Oncology Group (COG) AAML1031 phase III trial, 5-year IS was 15% for HR patients who relapsed versus 44% for initially low-risk patients who relapsed (*p* < 0.001) [45]. A meta-analysis comparing post-remission hematopoietic stem cell transplantation to chemotherapy alone in newly diagnosed HR pediatric AML reported a 31% improvement in disease-free survival [46]. However, concerns about transplant-related mortality and severe toxicities have led some studies to advise against routine post-remission transplantation [47]. Given the conflicting data, larger prospective studies are required.

The response to induction therapy at the initial diagnosis significantly affects survival in patients with relapsed AML. Patients without bone marrow response after first induction exhibit a 5-year OS approaching 0%, than 45% in responders [48]. Similarly, in the COG cohort, 5-year OS was 24% in patients with persistent minimal residual disease (MRD) post-induction versus 41% in MRD-negative cases. In the present study, patients with poor initial induction response exhibited reduced OS. The BFM trials reported 5-year OS of 29% in patients relapsing within 12 months of diagnosis versus 55% in later relapses, consistent with our observation of lower OS in patients with a shorter time-to-relapse [45]. It has been observed that patients whose risk group was determined according to the MRD response during induction therapy and who received mitoxantrone during the first induction had higher 5-year EFS and OS rates [2]. In our study, MRD could not be measured in our patients and mitoxantrone was administered during the second induction. Since MRD could not be measured, HR patients in our study may have been treated as IR or SR. If the MRD responses of these patients could have been evaluated, EFS and OS rates could have been increased in the high-risk group through stem cell transplantation.

Our center began performing BMT in 2017; therefore, the number of patients who underwent BMT was small. While BMT carries risks such as graft-versus-host disease and infections, it remains a potential curative alternative for HR and relapsed AML [49]. Post-BMT survival is inversely related to time-to-relapse, with OS reducing to 4% in patients relapsing within 6 months post-transplant versus 23% in those relapsing after 1 year [50]. In our cohort, only one HR patient underwent BMT without prior relapse; however, it succumbed to rapid post-transplant relapse. Survival outcomes in BMT recipients may also vary according to cytogenetic profile [51]. Survival rates among our transplant patients were lower than those reported in the literature, likely reflecting the small sample size. Mortality risk in transplanted AML patients is closely linked to the time between transplant and relapse; the shorter this interval, the poorer the survival [50]. We obtained good BMT outcomes in patients with HR who remained in continuous remission, whereas responses were extremely poor in those who relapsed before or after transplantation.

The limitations of the present study include its retrospective design, low number of patients and the lack of MRD assessment in AML patients in our country, which precluded survival analyses based on genetic findings due to inadequate data.

In the present study, the key factors influencing survival in pediatric AML patients included risk group, age at diagnosis, induction response at diagnosis, and time-to-relapse. Among the relapsed patients, survival was also affected in the initial risk group. Leukapheresis did not affect survival. Mortality remained high in non-AML M3 cases. Further research is required to develop genetically defined treatment subgroups for pediatric patients with AML. We recommend more stringent risk stratification for IR patients under the AML-BFM 2012 protocol and advocate larger studies aimed at creating standardized, personalized treatment protocols for all patients.

## Data Availability

The data that support the findings of this study are available upon request from the corresponding author. The data is not publicly available because of privacy or ethical restrictions.
